# Plasma-Free Blood as a Potential Alternative to Whole Blood for Transcriptomic Analysis

**DOI:** 10.1007/s43657-023-00121-1

**Published:** 2023-09-13

**Authors:** Qingwang Chen, Xiaorou Guo, Haiyan Wang, Shanyue Sun, He Jiang, Peipei Zhang, Erfei Shang, Ruolan Zhang, Zehui Cao, Quanne Niu, Chao Zhang, Yaqing Liu, Leming Shi, Ying Yu, Wanwan Hou, Yuanting Zheng

**Affiliations:** 1 State Key Laboratory of Genetic Engineering, School of Life Sciences and Human Phenome Institute, Shanghai Cancer Center, Fudan University, Shanghai, 200438 China; 2 Shandong Provincial Hospital, Shandong First Medical University, Jinan, 250021 China; 3 The International Human Phenome Institutes, Shanghai, 200438 China

**Keywords:** RNA sequencing, Whole blood, Plasma-free blood, Serum-free blood, Transcriptomic analysis

## Abstract

RNA sequencing (RNAseq) technology has become increasingly important in precision medicine and clinical diagnostics, and emerged as a powerful tool for identifying protein-coding genes, performing differential gene analysis, and inferring immune cell composition. Human peripheral blood samples are widely used for RNAseq, providing valuable insights into individual biomolecular information. Blood samples can be classified as whole blood (WB), plasma, serum, and remaining sediment samples, including plasma-free blood (PFB) and serum-free blood (SFB) samples that are generally considered less useful byproducts during the processes of plasma and serum separation, respectively. However, the feasibility of using PFB and SFB samples for transcriptome analysis remains unclear. In this study, we aimed to assess the suitability of employing PFB or SFB samples as an alternative RNA source in transcriptomic analysis. We performed a comparative analysis of WB, PFB, and SFB samples for different applications. Our results revealed that PFB samples exhibit greater similarity to WB samples than SFB samples in terms of protein-coding gene expression patterns, detection of differentially expressed genes, and immunological characterizations, suggesting that PFB can serve as a viable alternative to WB for transcriptomic analysis. Our study contributes to the optimization of blood sample utilization and the advancement of precision medicine research.

**Supplementary Information:**

The online version contains supplementary material available at 10.1007/s43657-023-00121-1.

## Introduction

RNA sequencing (RNAseq) technology (Wang et al. 2009; Stark et al. 2019) for transcriptomic analysis has become increasingly important in the fields of precision medicine (De Ruysscher et al. 2017) and clinical diagnostics (Ruan et al. 2021). RNAseq has also emerged as a powerful tool for identifying protein-coding genes (Wang et al. 2009; Hong et al. 2019), performing differential expression gene (DEG) analysis for signature discovery (Soneson et al. 2015), and inferring immune cell composition (Newman et al. 2015). Human peripheral blood samples are widely used for RNAseq due to their availability and convenience of collection (Mohr and Liew 2007; Qi et al. 2023), offering valuable insights into the biomolecular information of the individual. As the transcriptome of blood samples has been intensively studied (Hong et al. 2019), it has been shown that it can help improve the diagnosis of patients with rare diseases and identify new disease-related genes (Fresard et al. 2019). The analysis of blood transcriptome can provide prospective scientific guidance for disease prevention, diagnosis (de Almeida Chuffa et al. 2022), treatment (He et al. 2019b), and prognosis (Krishnan and Thomas 2022).

Blood samples can be classified into whole blood (WB), plasma, serum, blood cells, and other remaining sediment samples according to post-collection processing (Bayot and Tadi 2022). Plasma samples are obtained by centrifugation of WB samples with anticoagulants, while serum samples are obtained by centrifugation of WB samples without anticoagulants or with procoagulants (Sotelo-Orozco et al. 2021). WB, plasma, and serum samples are commonly used for transcriptomic studies due to their sample availability and convenience of collection under ideal conditions (Qin et al. 2016; Mjelle et al. 2019; Husseini et al. 2022). However, the remaining sediment samples after plasma or serum separation are often discarded or stored for biological resource preservation in real-world scenarios. These include plasma-free blood (PFB) and serum-free blood (SFB) samples, which also contain abundant RNAs that reflect individual information. WB samples contain various immune cell types, such as peripheral blood mononuclear cells (PBMCs), polymorphonuclear cells (granulocytes), and other anucleate cells (e.g., erythrocytes and platelets) according to the number of cell nuclei (Uhlen et al. 2019). PFB samples are like WB samples but without plasma. Moreover, the cells in SFB samples are all enclosed in a blood clot, which may lead to differences in their expression when compared to WB and PFB samples.

For retrospective cohort studies that rely on biobank WB samples from authoritative institutions and hospitals for transcriptomic research, PFB and SFB samples may serve as alternative sources of RNA for sequencing when WB samples are insufficient. Nevertheless, the feasibility of using PFB and SFB samples for transcriptome analysis remains unclear. Moreover, as multi-omics studies such as genomics, transcriptomics, proteomics, metabolomics, and microbiomics require adequate blood samples (Wu et al. 2021a), it is essential to maximize the utilization of blood samples including PFB and SFB for identifying biomarkers in the blood for achieving personalized treatment (De Ruysscher et al. 2017; Kamali et al. 2022).

Given these concerns, our study aimed to assess the suitability of employing either PFB or SFB samples as alternative RNA sources in transcriptomic analysis. Previous researches have demonstrated the influence of pre-analytical factors on RNA quality and gene expression (Debey-Pascher et al. 2011; Mastrokolias et al. 2012; Dvinge et al. 2014; Shin et al. 2014; Zhao et al. 2014; Huang et al. 2017; Reust et al. 2018; Shen et al. 2018; Donohue et al. 2019; Gautam et al. 2019; He et al. 2019a; Harrington et al. 2020; Xing et al. 2021; Chebbo et al. 2022; Husseini et al. 2022). However, a comprehensive understanding of the similarities and differences among WB, PFB, and SFB samples in transcriptomic profiles remains elusive, making it crucial to evaluate their suitability for various applications using reliable performance metrics.

In the comparative study, we analyzed the transcriptomic profiles of WB, PFB, and SFB samples collected from two healthy donors at two different holding times. We assessed expression patterns, differentially expressed genes (DEGs), and immunological characterizations for each blood type. Our results indicate that PFB samples may be used as a potential alternative to WB samples for transcriptomic analysis, because they exhibit expression patterns that closely resemble to those of WB samples based on various performance metrics. This would provide a valuable resource for maximizing blood sample utilization in research and clinical applications. Further research is needed to confirm the suitability of PFB and SFB samples for various specific applications.

## Methods

### Blood Sample Collection and Preparation

Informed consents were obtained from two healthy donors (P10: male; P11: female) by following the study protocol approved by the Ethics Committee of Fudan University. We collected 5 mL of blood from each donor using anticoagulation tubes with K2-Ethylenediaminetetraacetic acid (EDTA) (Shanghai Aoxiang Medical Technology Co., Ltd.) and procoagulation tubes (Jiangsu Yuli Medical Instruments Co., Ltd.). One group of blood samples was processed after holding 0.5 h (H0) and another group was processed after holding 6 h (H6) at room temperature to simulate the real turnaround times. PFB samples were obtained by centrifuging blood samples collected in anticoagulation tubes and separating the plasma supernatants, while SFB samples were obtained by centrifuging blood samples collected in procoagulation tubes and separating the serum supernatants. For each donor, three tubes with 150 µL WB samples, three tubes with 100 µL PFB samples, and one tube with 1.20 mL SFB samples were used for RNA extraction, and the remaining samples were snap frozen in liquid nitrogen and stored at – 80 ℃ in the refrigerator. The separated plasma and serum samples in this study were used in another parallel study for miRNA analysis using small RNA sequencing.

### RNA Extraction and cDNA Library Construction

RNA samples were extracted by QIAzol lysis reagents (QIAGEN) with QIAcube Connect (QIAGEN) according to the manufacturer's manual. RNA concentration and integrity were measured by Qubit 3.0 Fluorometer (Thermo Fisher Scientific) and Agilent 4200 TapeStation (Agilent Technologies (China) Co., Ltd.), respectively. We normalized RNA concentrations according to the volume ratio of PFB vs WB samples and SFB vs WB samples to make them comparable among different types of blood samples. RNA samples with a total RNA amount ≥ 100 ng and RNA integrity number (RIN) score ≥ 3 were qualified to construct Ribozero RNAseq libraries for sequencing (Li et al. 2014).

Globin and ribosomal RNAs were depleted using Ribo-off Globin & rRNA Depletion Kit (H/M/R) (Vazyme #N408), and cDNA libraries were constructed using VAHTS® Universal V8 RNAseq Library Prep Kit for Illumina (Vazyme #NR605). The concentrations of cDNA libraries were measured using a Qubit 3.0 Fluorometer (Thermo Fisher Scientific). The distribution of cDNA fragment size was measured by Qsep 100 Advance (BiOptic).

### RNA Sequencing and Data Preprocessing

cDNA libraries were sequenced on the NovaSeq 6000 sequencing platform (Illumina) to generate paired-end reads (150 bp). Then we followed a previously published protocol (Chen et al. 2022) for processing and quality control of raw FASTQ reads. Briefly, adapter sequences were trimmed using fastp v0.19.6 (Chen et al. 2018) and read quality was assessed, while after trimming using FastQC v0.11.5 (https://www.bioinformatics.babraham.ac.uk/projects/fastqc/). Potential contamination from other species or junction primers was detected by extracting the first 10,000 reads from the clean FASTQ files using FastQ Screen v0.12.0 (Wingett and Andrews 2018). The mapping quality of 10% bam file per sample was calculated using Qualimap v2.0.0 (Okonechnikov et al. 2016) for efficiency and cost-effectiveness purposes. Reads were aligned to GRCh38_snp_tran genome reference and quantified using HISAT v2.1 (Kim et al. 2019), SAMtools v1.3.1 (Li et al. 2009), and StringTie v1.3.4 (Pertea et al. 2015) pipeline (Pertea et al. 2016) with Ensembl gene models (version: Homo_sapiens.GRCh38.93.gtf).

Gene expression was normalized by fragments per kilobase of exon model per million mapped fragments (FPKM) and transcripts per kilobase of exon model per million mapped reads (TPM) to remove the effect of gene length and library size (Zhao et al. 2021). The counts were used for correlation analysis and to define detected genes for the Jaccard index (Mukherjee et al. 2021) comparison, principal variance component analysis (PVCA), as well as DEG analysis. FPKM was used for coefficient of variation (CV) calculation, sex check, principal component analysis (PCA), and hierarchical clustering analysis (HCA). TPM values were deconvoluted to infer the proportion of immune cell expression. Only protein-coding genes were used for CV calculation, PCA, and correlation analysis. To avoid infinite values, a minimum value of 0.01 was added to the FPKM value of each gene before the log2 transformation.

### Sex-Specific Gene Expression and Sex Check

Sex check was performed using sex-specific genes identified from previous studies (Chen et al. 2022), including five male-specific genes (*RPS4Y1*: *Ribosomal Protein S4 Y-Linked 1*, *DDX3Y*: *DEAD-Box Helicase 3 Y-Linked*, *EIF1AY*: *Eukaryotic Translation Initiation Factor 1A Y-Linked*, *KDM5D*: *Lysine Demethylase 5D*, *TXLNGY*: *Taxilin Gamma Pseudogene*, *Y-Linked*) and two female-specific genes (*XIST*: *X Inactive Specific Transcript*, *TSIX*: *TSIX Transcript*, *XIST Antisense RNA*). A distance matrix using the Euclidean method was calculated to measure the distance of the samples and genes, whereas Ward linkage was used for HCA using the R package pheatmap v1.0.12.

### Human Protein-Coding Gene Detection in Different Blood Types

Similarities in protein-coding gene expression of all samples were compared using PCA. PCA was conducted with the univariance scaling, using the prcomp function of R package stats v4.2.1. Moreover, the clustering of protein-coding genes in the top 1000 standard deviation (SD) ranking across all samples was compared using HCA. HCA was performed using the R package pheatmap v1.0.12 with clustering based on a distance matrix calculated using the correlation distance metric. PVCA was performed using the R packages pvca v1.36.0 and Biobase v2.56.0 to assess the impact of different factors on the SD TOP 1000 protein-coding gene expression data. Pearson correlation coefficient (PCC) was calculated for the intra-group and inter-group to show correlations between technical replicates vs correlations between different blood types. The detected genes of the individual sample were defined as the genes with ≥ three counts in ≥ two technical replicates of three. The similarity of detected protein-coding gene sets between different groups was evaluated using the Jaccard index (Mukherjee et al. 2021). Finally, the detected protein-coding genes were filtered and compared between blood types with the same donor and the same holding time using R package eulerr v6.1.1 to produce Venn diagrams.

### Differential Expression Analysis and Enrichment Analysis

The R package limma v3.52.4 (Ritchie et al. 2015) was used to identify DEGs with a *p* < 0.05 and | log2FC |≥ 1. Venn diagrams created by the R package eulerr v6.1.1 were used to compare and visualize the results. Gene Ontology (GO) and Kyoto Encyclopedia of Genes and Genomes (KEGG) enrichment analysis were performed using the R package ClusterProfiler 4.0 v4.4.4 (Roncaglia et al. 2017; Wu et al. 2021b) and KOBAS-I (http://kobas.cbi.pku.edu.cn/genelist/) (Bu et al. 2021) to assess the enrichment of DEGs in each group based on blood types or donors as variables.

### Immune Cell Cluster Characterization and Cell Abundance

The cell-type identification by estimating relative subsets of RNA transcripts (CIBERSORT) algorithm (Newman et al. 2015) was used to estimate the cell-type composition of each sample using the R package IOBR v0.99.9 (Zeng et al. 2021), which translated the TPM-normalized gene expression matrix of different blood types into the relative proportion of immune cells. Euclidean distance was performed to assess the correlations between immune cell subsets using R package pheatmap v1.0.12.

### Immune Cell-Specific Gene Expression

The top 60 genes specifically expressed in six major cell types of human blood (granulocytes, monocytes, dendritic cells, nature killer (NK) cells, B cells, and T cells) were collected from the "IMMUNE CELL" section of the website for Human Protein Atlas (HPA) (Uhlen et al. 2019), which can be accessed at https://www.proteinatlas.org/humanproteome/immune+cell. TPM values of these genes were used for cluster and comparative analysis in different blood types.

### Statistical Analysis

All experiments were performed with three replicates. Statistical analysis and graphical work were performed using R v4.2.1 (https://cran.r-project.org/, R development core team) and a suite of R packages. Student's *t* test and Wilcoxon test were used to compare RNAseq quality control metrics and gene expression profiles between groups with different blood holding times, donors, and blood types. All statistical tests were two tailed, and a *p* value less than 0.05 was considered statistically significant.

## Results

### Study Overview

**Fig. 1:**
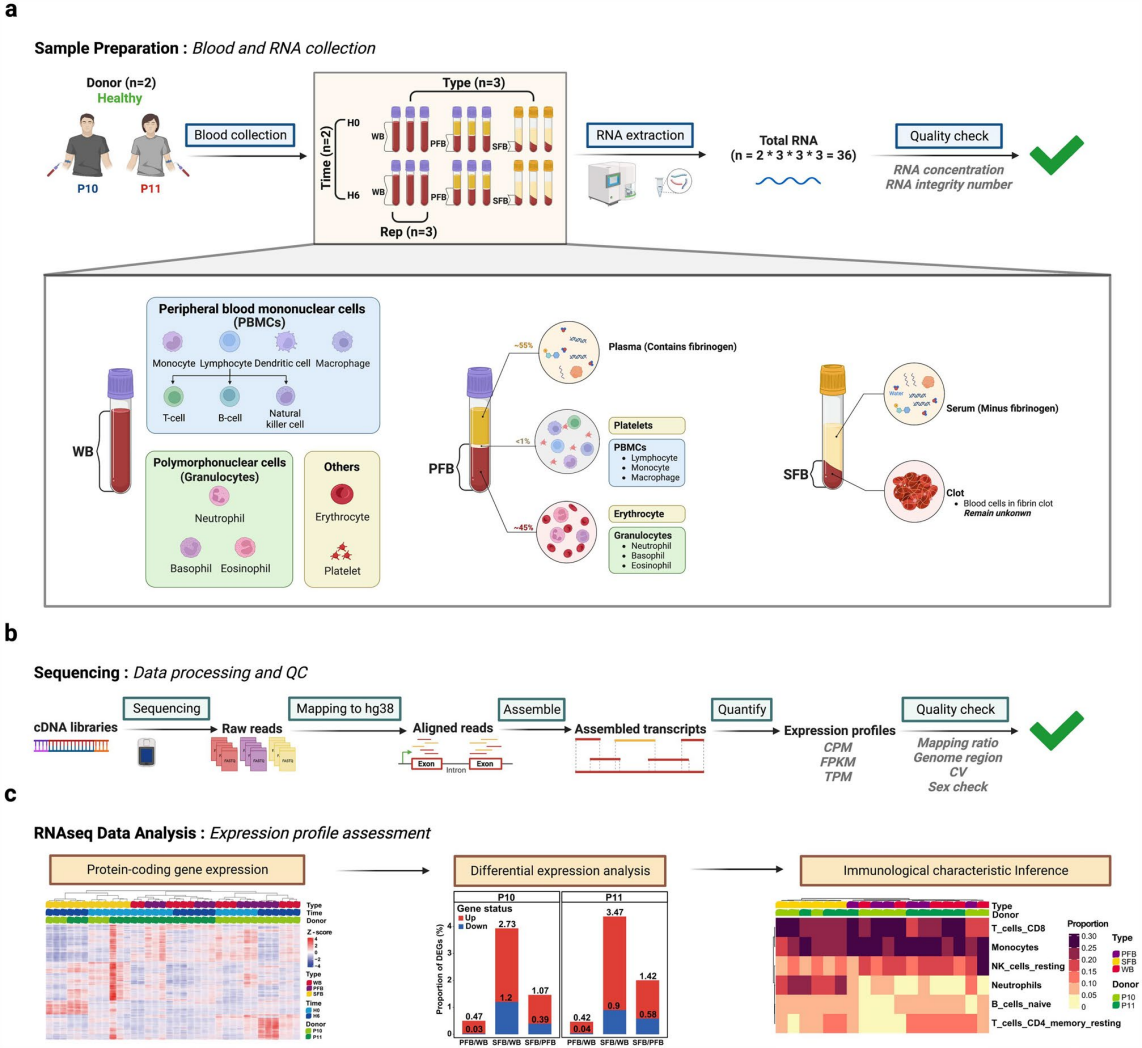
Overview of study design and analysis. **a** Top: Sample collection and processing. Whole blood (WB), plasma-free blood (PFB), and serum-free blood (SFB) samples were collected from two healthy donors (P10 and P11) through two holding times (H0 and H6). (P10: male donor, P11: female donor. H0: processed after holding 0.5 h, H6: processed after holding 6 h.) Bottom: Diagram of estimated immune cell composition in three blood sample types. Total RNA was extracted from each blood sample and assessed for quality, including RNA concentration and RNA integrity number (RIN). **b** RNA sequencing and quality control. The RNA samples were subjected to library preparation and sequencing. The sequencing data were aligned to the hg38 reference genome. Transcript abundance was estimated for each sample, and the quality check was performed using various metrics, including mapping ratio, genome region, coefficient of variation, and sex check. **c** Bioinformatic analysis of RNAseq data. The expression of protein-coding genes across all samples was shown by HCA. Differential expression gene analysis was conducted between different blood types and donors. Immune cell composition was inferred for each blood type using gene expression data

In this study, we performed a comparative transcriptomic analysis of three blood types, including WB, PFB, and SFB, collected from two healthy donors at two different holding times (Fig. 1a, top). WB samples consist of diverse immune cell types, including PBMCs, polymorphonuclear cells (granulocytes), as well as anucleate cells (e.g., erythrocytes and platelets) based on their number of cell nuclei. PFB samples are similar to WB samples, while the cells in SFB samples are all enclosed in a blood clot (Fig. 1a, bottom). The RNA samples were subjected to library preparation and sequencing (Fig. 1b). The sequencing data were aligned to the hg38 reference genome, and transcript abundance was estimated for each sample. The quality check was performed using various metrics, including mapping ratio, genome region, CV, and sex check. To compare expression profiles across blood types, we employed various analytical methods such as PCA, HCA, PVCA, PCC, and the Jaccard index. Additionally, we identified DEGs and compared immunological characterizations across the three blood types using different groups (Fig. 1c).

### High-Quality RNA and Sequencing Data Obtained from Each Blood Sample

**Fig. 2:**
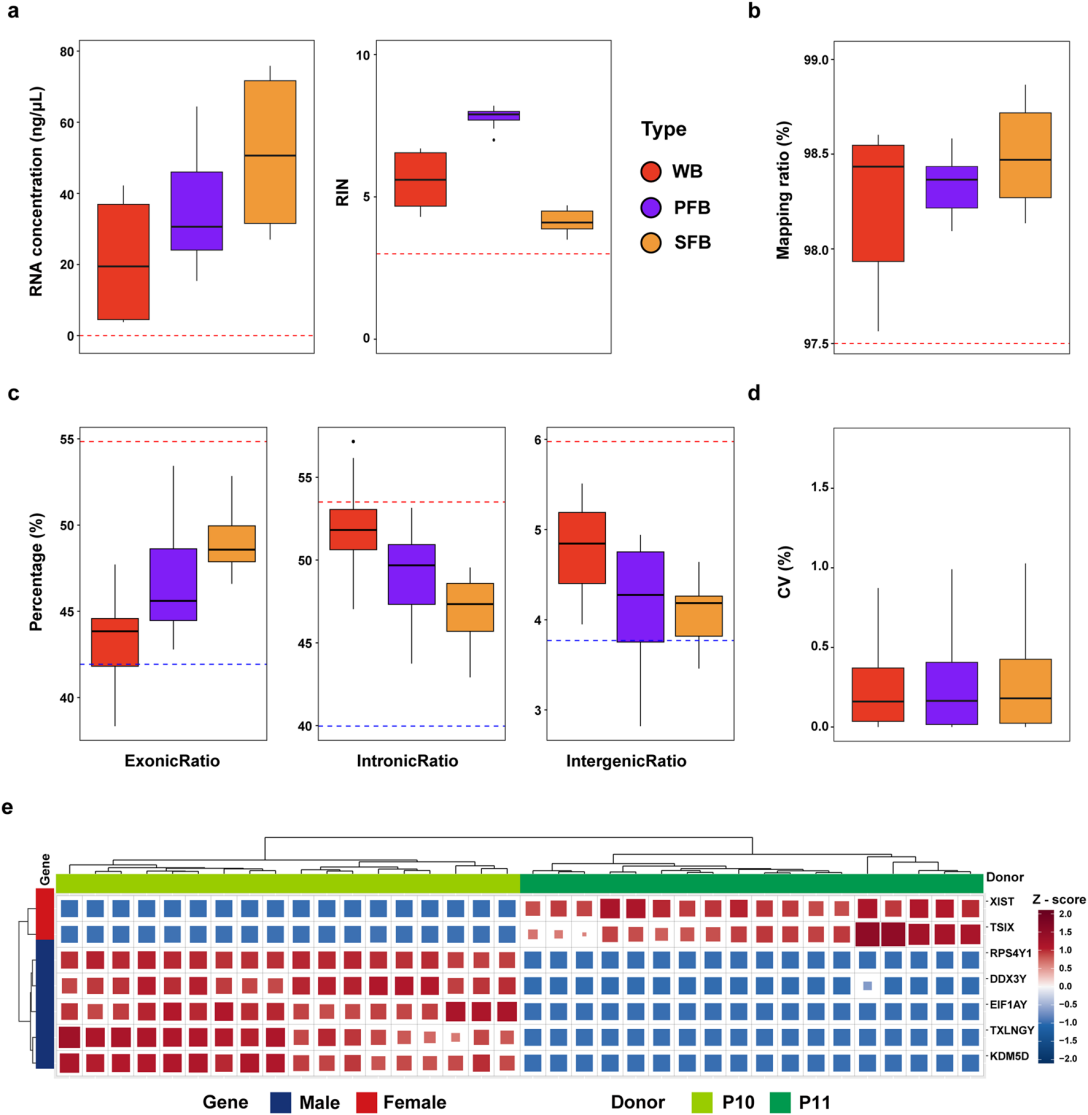
Quality assessment of RNAseq data. **a** RNA concentrations (ng/μL) and RIN values in three types of blood samples (left red line value: 1; right red line value: 3). **b** The mapping ratio (%) in three types of blood samples (The red line represents the median value (97.51%) corresponding to the RiboZero library mapping ratio in the Quartet dataset). **c** The distribution of reads across the human genome. The dotted line represents the mean ± SD values of the corresponding gene regions of the RiboZero library in the Quartet RNA reference dataset. Red indicates mean + SD value (ExonicRatio: 54.85, IntronicRatio: 53.51, and IntergenicRatio: 5.98), and blue indicates mean − SD value (ExonicRatio: 41.93, IntronicRatio: 39.97, and IntergenicRatio: 3.77). **d** The coefficient of variation (CV) values of protein-coding gene expression in three blood types** e** Heatmap and hierarchical clustering of the expression levels of seven sex-specific genes in all samples, including five male-specific genes (*RPS4Y1*, *DDX3Y*, *EIF1AY*, *KDM5D*, and *TXLNGY*) and two female-specific genes (*XIST* and *TSIX*). Blue indicates male-specific genes, and red indicates female-specific genes. Lime green indicates P10, and dark green indicates P11

We assessed RNA quality by measuring RNA concentration and RIN score for each sample. The minimum RNA concentration required for cDNA library construction was 1 ng/μL (Li et al. 2014). All samples had RNA concentrations above this threshold with a median value of 34.50 ng/μL (Fig. 2a, left; Table 1). Furthermore, there was no significant difference in normalized RNA concentrations between the WB and SFB samples (Fig. S1). Similarly, the minimum RIN score required for sequencing was three (Li et al. 2014). All samples had RIN scores above this threshold, with a median value of 5.6 (Fig. 2a, right; Table 1). These results indicated that all samples were suitable for RiboZero library construction and sequencing.

**Table 1:** RNA quantity and quality

Donor	Time	Type	Conc	RIN
P10	H0	WB	35.30 ± 5.13	4.50 ± 0.17
P10	H0	PFB	60.27 ± 6.47	7.27 ± 0.23
P10	H0	SFB	74.87 ± 2.84	5.82 ± 1.09
P10	H6	WB	40.47 ± 3.04	5.86 ± 0.47
P10	H6	PFB	39.07 ± 5.14	7.97 ± 0.12
P10	H6	SFB	69.13 ± 6.60	5.89 ± 1.12
P11	H0	WB	6.00 ± 1.92	5.57 ± 1.03
P11	H0	PFB	21.93 ± 5.40	7.87 ± 0.06
P11	H0	SFB	31.33 ± 2.89	5.85 ± 1.02
P11	H6	WB	7.19 ± 3.42	6.60 ± 0.10
P11	H6	PFB	23.00 ± 5.55	8.07 ± 0.12
P11	H6	SFB	34.07 ± 5.75	4.66 ± 1.76

Conc. RNA concentration (ng/μL), RIN RNA integrity number

The average mapping ratio of all samples was 98.36% (Fig. 2b), all above the median mapping ratio of 97.51% for the RiboZero libraries in the Quartet RNA reference datasets (Yu et al. 2023), which suggested the successful alignment of the reads to the reference genome. The gene region distribution was consistent with the expected characteristics of RiboZero libraries in the Quartet RNA reference datasets (Yu et al. 2023) across the three blood types (Fig. 2c). The median CV values of the sample replicates for the three blood types were all less than 0.17%, indicating good reproducibility of expression data (Fig. 2d). Additionally, a sex check was performed to validate the gender of the sample donors. Specifically, seven sex-specific genes were analyzed, including five male-specific genes and two female-specific genes. The results of the sex check were found to be consistent with the expected gender of each donor (Fig. 2e), thus affirming the quality of our expression data and providing confidence in the subsequent analysis.

Moreover, we performed additional quality control analysis on various metrics of the raw and mapped data (Fig. S2). Our findings revealed that: (1) high Phred quality scores were observed across all bases and positions in the FASTQ files (Fig. S2a); (2) no contamination from extraneous sources was detected in any of the samples (Fig. S2b); (3) the GC content exhibited a normal distribution in all samples (Fig. S2c). Details of the quality control metrics are provided in Table S1. Overall, high-quality RNA and sequencing data could be obtained from each blood type, by employing rigorous quality control measures. These results established a strong foundation for further investigation into comparative gene expression profiling.

### Consistent Expression of Human Protein-Coding Genes in PFB and WB Samples

**Fig. 3:**
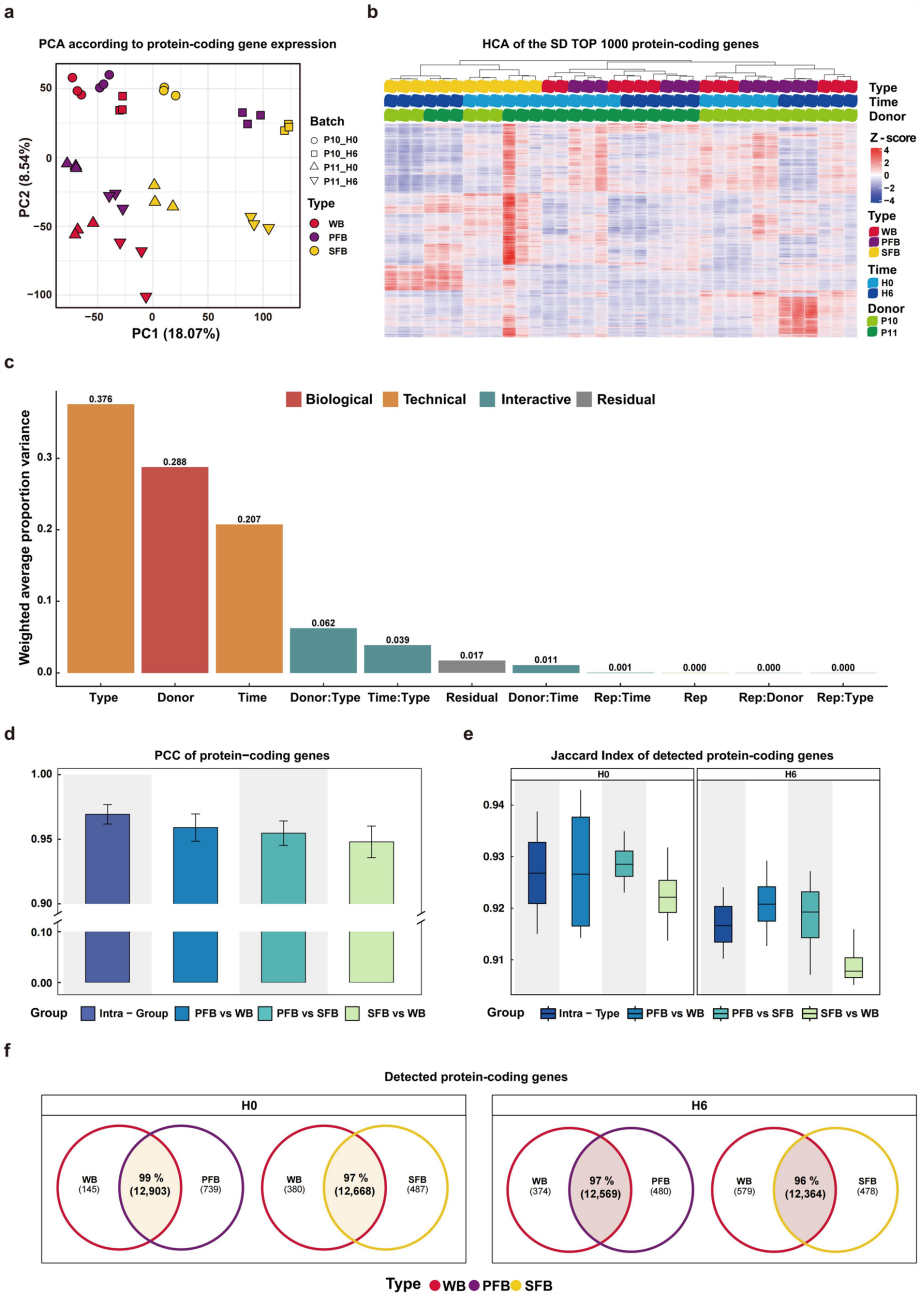
Human protein-coding gene expression analysis. **a** Principal component analysis (PCA) based on FPKM values of protein-coding genes in each sample (red represents WB, purple represents PFB, and yellow represents SFB. The shape indicates the donor and the holding time of each sample.). **b** Heatmap of the top 1000 most variable (SD top 1000) protein-coding genes across all samples, selected and normalized by genes. “Type” represents the blood type, “Donor” represents the donor, and “Time” represents the holding time. **c** Principal variance component analysis (PVCA) based on log2-transformed CPM values (+ 0.5) of the top 1000 SD protein-coding genes across all samples. **d** Barplot of the Pearson correlation coefficients for the protein-coding gene expression profiles (counts) in different sample groups, with the intra-group indicating the same blood type at different holding times. **e** Boxplot of Jaccard index of protein-coding genes expression levels (counts) in different groups at H0 and H6, with intra-type indicating samples from the same blood type in the same holding time. **f** Venn diagram of the number of detected expression genes in different blood types at H0 and H6

Due to the limited sequencing depth of our data, we primarily focused on the examination of highly expressed protein-coding genes as compared to non-coding genes in our expression analysis to ensure the reliability of our findings. We performed PCA of the protein-coding gene expression profiles to evaluate the effects of the donor, holding time, and blood type. The result showed that data were primarily separated by blood type on the first principal component (PC1) and by the donor on the second principal component (PC2), while samples from the same donor and holding time were clustered together by blood type (Fig. 3a). Notably, the PFB and WB samples of donor P10 from H0 were more closely related to each other than to other samples, and the same was true for the PFB and WB samples of donor P11, indicating a higher similarity between PFB and WB samples. To further investigate this similarity, we performed HCA on the SD TOP 1000 protein-coding genes in each sample, revealing that PFB and WB samples clustered together, whereas SFB samples clustered separately (Fig. 3b). Additionally, we employed PVCA to evaluate the contribution of different factors to transcriptional profile heterogeneity. The main factor was blood type (technical factor), followed by the donor (biological factor) and holding time (technical factor) (Fig. 3c).

Then PCC was calculated for the protein-coding gene expression profiles (counts) in different sample groups. The expression levels of protein-coding genes were highly correlated across blood types, where the correlations between PFB and WB samples were closest to those among technical replicates (positive control: Intra-Group) (Fig. 3d). Detailed correlation analysis for all samples is presented in Fig. S2. Additionally, the Jaccard index was used to measure the consistency of gene detection among groups. Our analysis revealed that all groups had Jaccard index values above 0.91 (Fig. 3e) and that the Jaccard index values between PFB and WB samples were closest to those between technical replicates, indicating the similarity in protein-coding gene detection between PFB and WB samples. Last, we employed Venn diagrams to compare the number and proportion of detected protein-coding genes among WB, PFB, and SFB samples at H0 and H6. The results showed that over 96% of the protein-coding genes were detectable in PFB vs WB samples and SFB vs WB samples (Fig. 3f) at H0 and H6, demonstrating that each blood type was amenable to human protein-coding gene detection.

Above all, we demonstrated the consistent expression of human protein-coding genes in PFB and WB samples, highlighting their high similarity. However, to exclude the effect of holding time on our main findings (Fig. 3c), we only selected samples from H0 in subsequent analysis.

### Less Variation Between PFB and WB Samples in Differential Expression Analysis

**Fig. 4:**
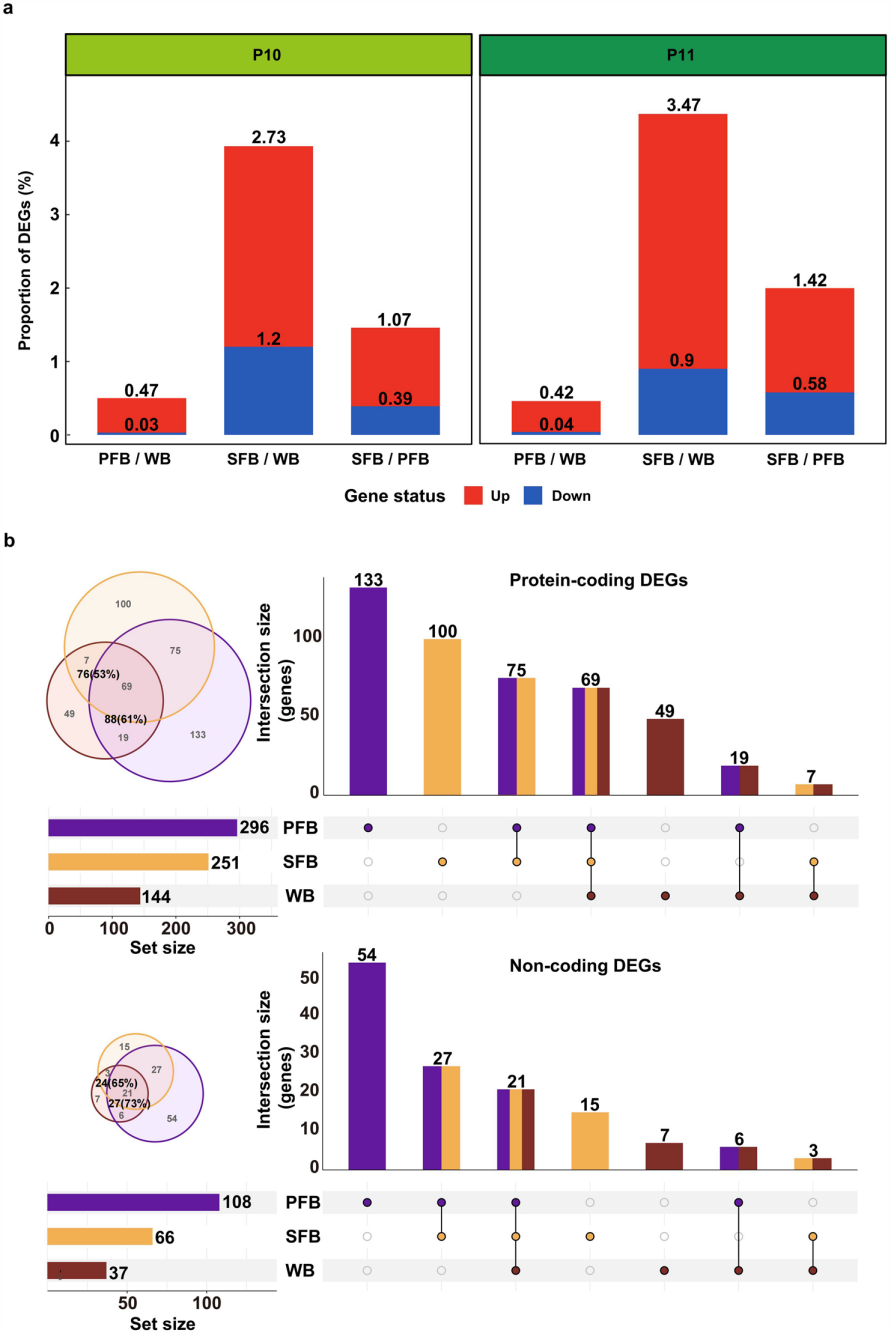
Differential expression genes analysis in different groups. **a** The percentage of differentially expressed genes (DEGs) between different blood samples from two donors. The comparisons are PFB vs WB, SFB vs WB, and SFB vs PFB. Red indicates significantly upregulated genes, and blue indicates significantly downregulated genes. **b** The number of DEGs between two different donors for the three blood types. The comparisons are P10_WB vs P11_WB, P10_PFB vs P11_PFB, and P10_SFB vs P11_SFB. The Venn diagram and upset plot provide an overview of the number of DEGs from the three blood types, including both protein-coding (up) and non-coding DEGs (down)

To investigate the utility of the three blood types, we conducted a DEG analysis on WB, PFB, and SFB samples derived from two donors (P10 and P11) across three blood type comparisons (PFB vs WB, SFB vs WB, SFB vs PFB). As expected, we found that the DEGs proportion between the three types of blood samples was low, ranging from a minimum of 0.46% (PFB vs WB in P11) to a maximum of 4.37% (SFB vs WB in P11) of all detected genes. Specifically, we observed that the smallest proportion of DEGs was observed between PFB and WB samples, accounting for approximately 0.5% (Fig. 4a), which further indicated that PFB and WB samples may have similar gene expression profiles.

Moreover, we identified the DEGs between the two donors across three blood types (P10_WB vs P11_WB, P10_PFB vs P11_PFB, and P10_SFB vs P11_SFB) to assess the ability to detect biological differences. To determine the extent of overlap between DEGs, we compared PFB and SFB with WB samples. PFB samples covered 61% (88 in PFB / 144 in WB) DEGs of protein-coding genes and 73% (27 in PFB / 37 in WB) DEGs of non-coding genes in WB samples, while SFB samples can cover 53% (76 in SFB / 144 in WB) DEGs of protein-coding genes and 65% (24 in SFB / 37 in WB) DEGs of non-coding genes in WB samples (Fig. 4b). The results showed that PFB samples shared more DEGs with WB than SFB did, indicating that PFB samples were closer to WB samples in terms of differential gene expression analysis. Generally, a limited number of DEGs are observed between healthy individuals, and these are typically not utilized in pathway enrichment. However, the enrichment analysis of DEGs in WB samples revealed that only one pathway (external side of plasma membrane) was significantly enriched between donors, suggesting that inter-individual differences in gene expression may not have significant biological implications. These findings suggested that PFB samples exhibited a greater similarity to WB samples in terms of differential gene expression analysis compared to SFB samples.

### Similar Properties of Immune Cell Expression in PFB and WB Samples

**Fig. 5:**
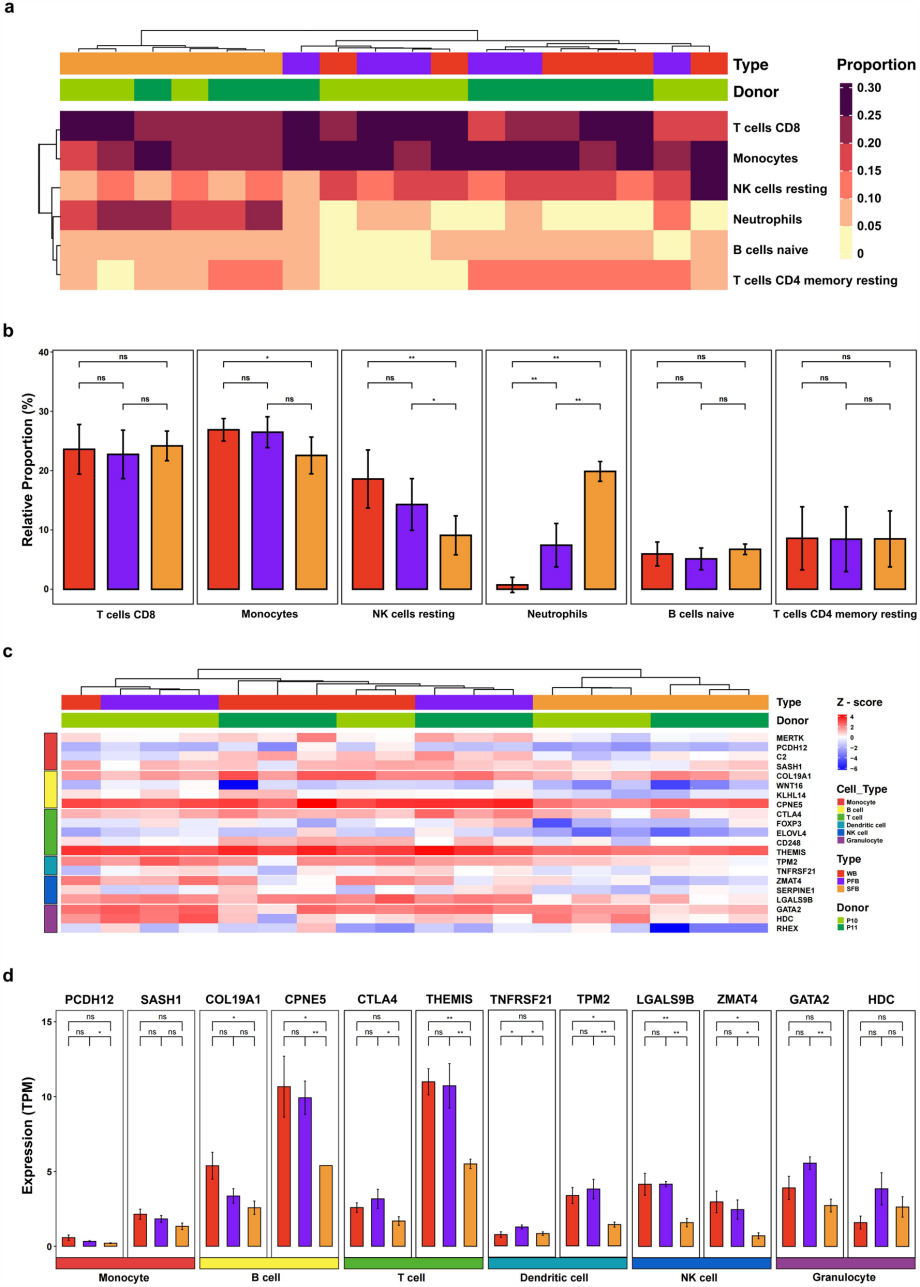
Inferred immunological characterizations. **a** Heatmap and hierarchical clustering of inferred relative abundance of immune cells in three blood types at H0 by CIBERSORT. Lime green indicates P10, and dark green indicates P11. **b** The proportion of six major immune cell types in each blood type at H0. **c** Heatmap and hierarchical clustering representing the expression levels of 21 immune cell-specific genes selected from the Human Protein Atlas (HPA) across six major immune cell types at H0 (pale peach: monocyte, deep pink: B_cell, magenta: T_cell, violet: dendritic_cell, dark purple: NK cell, black: granulocyte). **d** Comparison of expression levels of immune cell-specific genes between three blood types in six immune cell types

To compare the three blood types more comprehensively, we analyzed the composition of immune cells and the expression of related genes in WB, PFB, and SFB samples. The abundance of 22 infiltrating immune cells in each sample was estimated by CIBERSORT (Newman et al. 2015). We selected six cell types that had an average abundance of more than 1/22 across all samples for further analysis. HCA showed that WB and PFB samples clustered together while SFB samples formed a separate cluster (Fig. 5a). In addition, the inferred composition of immune cells in PFB samples showed greater similarity to that in WB samples, as compared to SFB samples, across the six immune cell types analyzed. The comparison between groups revealed that WB and PFB samples had no significant difference in immune cell composition except for neutrophils. SFB samples had higher proportions of neutrophils and lower proportions of resting NK cells than other samples (Fig. 5b). This may be due to the influence of platelets and other components in WB, PFB, and SFB samples that affected neutrophils content and expression (Ruf and Ruggeri 2010).

Furthermore, we collected 60 immune cell-related genes specific to major blood cell types from the HPA database. As different methods for classifying immune cells may vary, these six cell types of HPA may not entirely match the cell types from CIBERSORT results. After filtering out genes with unusually high or low expression levels across all samples, a subset of retained 21 genes was selected for subsequent clustering and comparative analysis. The clustering analysis produced consistent findings with the prior observations, signifying that WB and PFB samples also clustered together while SFB samples constituted a distinct cluster (Fig. 5c). Moreover, the comparative analysis between groups demonstrated that most immune cell-specific gene expressions were analogous between PFB and WB samples, whereas SFB samples exhibited certain discrepancies (Fig. 5d, Fig. S2). These results suggested that PFB and WB samples shared similar immunological characterizations.

## Discussion

Enhancing the utilization of blood samples can promote the advancement of precision medicine. In this study, we comprehensively compared transcriptomic profiles across three types of blood samples obtained from two healthy donors collected at two different holding times. The results implied that PFB samples may serve as a potential alternative to WB samples for specific applications where WB samples are impractical or unfeasible in retrospective and multi-omics studies. We confined the study to two donors to maintain the reproducibility of inter-individual biological differences. Our findings exhibited a high correlation between PFB and WB samples at the expression profile level, with fewer DEGs between PFB and WB samples compared to SFB and WB samples. Notably, the individual difference (donor) profiles also demonstrated greater similarity between PFB and WB samples than between SFB and WB samples. Besides, we showed that PFB and WB samples had analogous gene expression traits of immune cell proportion and immune cell-specific gene sets. These observations bore substantial biological relevance, underscoring the feasibility of employing PFB samples as a practical substitute for WB samples in investigations about immune functionality and disease research (Feng et al. 2022). In summary, PFB samples could serve as an alternative to WB samples for RNAseq, thereby augmenting the significance of PFB samples in biobanks.

The reason for more overlapping DEGs between two donors for PFB and WB samples (Fig. 4b) could be the higher number of DEGs in PFB samples compared to SFB samples. This discrepancy in DEG numbers stems from the fact that PFB samples have the highest number of detected genes (Fig. 3f). The increased number of detected genes in PFB samples can be attributed to the higher RIN score (Fig. 2a, right), indicating better RNA quality. The improved RNA quality in PFB samples enhances the sensitivity of gene detection, enabling the identification of more DEGs (Li et al. 2014). Additionally, only one pathway (external side of the plasma membrane) was significantly enriched between donors in WB samples, indicating that the inter-individual differences in gene expression between two healthy individuals might not carry substantial biological implications. Moreover, we identified the DEGs (Table S3) between WB and PFB samples for two donors and performed GO and KEGG pathway enrichment analysis. These common pathways are implicated in important biological processes, including immune regulation, cellular signaling, metabolic regulation, hormone synthesis, and action (Tables S4–S5). Further exploration of the regulatory mechanisms underlying these DEGs within specific pathways is crucial to enhance our understanding of the biological distinctions between PFB and WB samples. In summary, these differences may be due to the lack of plasma-specific components in PFB samples, such as liver-derived proteins or cytokines, which can contribute to the plasma-specific gene expression changes in WB samples (Benjamin and McLaughlin 2012; Rosenberg-Hasson et al. 2014; Liu et al. 2021). These results indicate the potential limitations of utilizing PFB samples as substitutes for WB samples in transcriptome analysis, particularly when investigating plasma-specific gene expression changes associated with altered immune responses or inflammatory conditions. Besides, discrepancies in neutrophil expression were observed between PFB and WB samples, suggesting that PFB samples might have certain limitations when employed as a surrogate for WB samples in the context of specific neutrophil-associated diseases (Wang et al. 2018; George et al. 2022; Gungabeesoon et al. 2023). Further investigation is required to clarify these differences and evaluate their potential consequences on the study of diseases about neutrophil function (Liew and Kubes 2019).

SFB samples are not recommended to be used as a substitute for WB samples in transcriptomic research unless they are the only available blood type. SFB samples may have differences in composition and cellular components compared to WB samples, which can affect the reliability and generalizability of the research findings. Additionally, in specific scenarios where PFB samples are considered as an alternative to WB samples, caution should be exercised when the research primarily focuses on cell-free molecules. PFB samples lack the plasma component, which may impact the analysis and interpretation of results related to cell-free molecules. Researchers should be mindful of these considerations to ensure the appropriate selection and utilization of blood samples in transcriptomic studies. Apart from that, the availability of a broader range of blood samples for transcriptomics, including PFB samples, may expand the opportunities for investigating gene expression in diverse contexts. Several alternative blood types, such as resected clots from large vessel acute ischemic stroke (Tutino et al. 2021), dried blood spots (Reust et al. 2018), placenta and umbilical cord blood from pregnant women and newborns (Lu et al. 2022), have demonstrated promise in transcriptome analysis, yielding valuable insights into disease mechanisms and pathways. Consequently, the continued investigation of these alternative blood types is of paramount importance.

It is important to acknowledge the limitations of this study, which are as follows. First, the study utilized a relatively modest sample size, comprising blood samples from merely two healthy donors, each with three technical replicates. To bolster the validity of our findings, we advocated for the enlargement of the participant pool in subsequent research endeavors, incorporating multiple paired-donor comparative analysis. Second, our analysis primarily focused on comparing transcriptomic results of PFB and WB samples from healthy donors, and we did not conduct a systematic analysis of the transcriptome specifically in the context of disease or stimulus for the blood samples. However, it is important to note that the extent to which various factors influence the similarity of transcriptomic analysis results between WB and PFB samples may vary depending on the specific disease, stimulus, and biological context. Further research is needed to comprehensively evaluate the utility and applicability of PFB samples in disease-related transcriptomic studies. Third, despite prior studies indicating that blood samples held for 6 h did not significantly impact RNA concentration (Kim et al. 2007) and integrity (Huang et al. 2017), our RNA expression profiles showed a discernible degree of differences between inter-type samples and intra-type samples held for 0.5 h and 6 h. The causal mechanism of this observation remains unclear and needs further investigation. Last, as transcriptome analysis of blood samples predominantly involved protein-coding genes (Wang et al. 2020), our study mainly focused on protein-coding genes, and the expression patterns of non-coding genes in different blood types remain to be studied.

## Conclusion

This study confers a substantial understanding pertaining to the feasibility of deploying PFB samples as a viable substitute for WB samples in RNAseq applications. The findings present crucial perspectives and potential approaches for increasing the use of infrequently collected blood samples in biospecimen repositories for retrospective studies and maximizing the utilization of blood samples in multi-omics cohort studies. Ultimately, the insights gleaned from this research contribute to the refinement of blood sample utilization in transcriptomic studies and the progression of precision medicine research.

### Supplementary Information

Below is the link to the electronic supplementary material.

Supplementary file1 (PDF 2704 KB)

Supplementary file2 (XLSX 15 KB)

Supplementary file3 (XLSX 29 KB)

Supplementary file4 (XLSX 28 KB)

Supplementary file5 (XLSX 14 KB)

Supplementary file6 (XLSX 15 KB)

## Data Availability

The blood samples, RNA materials, and datasets generated during the current study are available from the corresponding authors upon reasonable request.

## Abbreviations

RNAseq: RNA sequencing
WB: Whole blood
PFB: Plasma-free blood
SFB: Serum-free blood
PBMC: Peripheral blood mononuclear cell
DEG: Differential expressed gene
EDTA: Ethylenediaminetetraacetic acid
H0: Holding 0.5 h
H6: Holding 6 h
RIN: RNA integrity number
FPKM: Fragments per kilobase of exon model per million mapped fragments
TPM: Transcripts per kilobase of exon model per million mapped reads
PVCA: Principal variance component analysis
CV: Coefficient of variation
PCA: Principal component analysis
HCA: Hierarchical clustering analysis
RPS4Y1: Ribosomal protein S4 Y-linked 1
DDX3Y: DEAD-box helicase 3 Y-linked
EIF1AY: Eukaryotic translation initiation factor 1A Y-linked
KDM5D: Lysine demethylase 5D
TXLNGY: Taxilin gamma pseudogene, Y-linked
XIST: X inactive specific transcript
TSIX: TSIX transcript, XIST antisense RNA
SD: Standard deviation
PCC: Pearson correlation coefficient
GO: Gene Ontology
KEGG: Kyoto Encyclopedia of Genes and Genomes
CIBERSORT: Cell-type identification by estimating relative subsets of RNA transcripts
PC: Principal component
NK: Nature killer
HPA: Human protein atlas

## Code Availability

Our pipeline applications include all the Workflow Description Language (WDL, https://github.com/openwdl/wdl) processes required for RNAseq upstream analysis before the R analyses. Please note that the parameters in the pipeline applications are fixed, and the sample files can be directly processed. We have made the upstream analysis of RNAseq available on our GitHub repository (https://github.com/fudan-tnbc), and the dockers used in the upstream analysis can be obtained from Docker Hub (https://hub.docker.com/u/chenqingwang). The code used for pre-processing the upstream result data and drawing the figures can be found on our GitHub repository (https://github.com/xiaorouguo/c3bta). Quality control metrics for all data were collected in the metadata tables and visualized using R v4.2.1 (https://cran.r-project.org/, R development core team).
