# Supplementary material for: Plasma-Free Blood as a Potential Alternative to Whole Blood for Transcriptomic Analysis
Source: Phenomics. 2023 Sep 13;4(2):109–24. doi: 10.1007/s43657-023-00121-1 (PMC11169349; doi:10.1007/s43657-023-00121-1)
Supplement: Supplementary file 1 — Supplementary file1 (PDF 2704 KB) [file 43657_2023_121_MOESM1_ESM.pdf]

Supplementary Figure Legends

Fig S1. Normalized RNA concentration

Normalized RNA concentrations (ng/μL) in the three types of blood samples.

Fig S2. Quality control for clean data and sequence alignment

a Line plot displaying high-quality scores across all positions in the FASTQ file for each sample. **b** Box plot illustrating mapping ratio (%) for all samples, differentiated by source (red represents whole blood (WB) samples, purple represents plasma-free blood (PFB) samples, and yellow represents serum-free blood (SFB) samples). **c** GC content for the three types of blood samples.

Fig S3. Pearson correlation coefficient analysis

Heatmap representing the Pearson correlation coefficients (PCC) for the protein-coding genes expression profiles ($\log_2$(counts + 1)) across all samples.

Fig S4. Expression of 21 immune cell-specific genes

Comparison of expression levels of 21 immune cell-specific genes among three blood types, spanning six immune cell categories.

Supplementary Figures

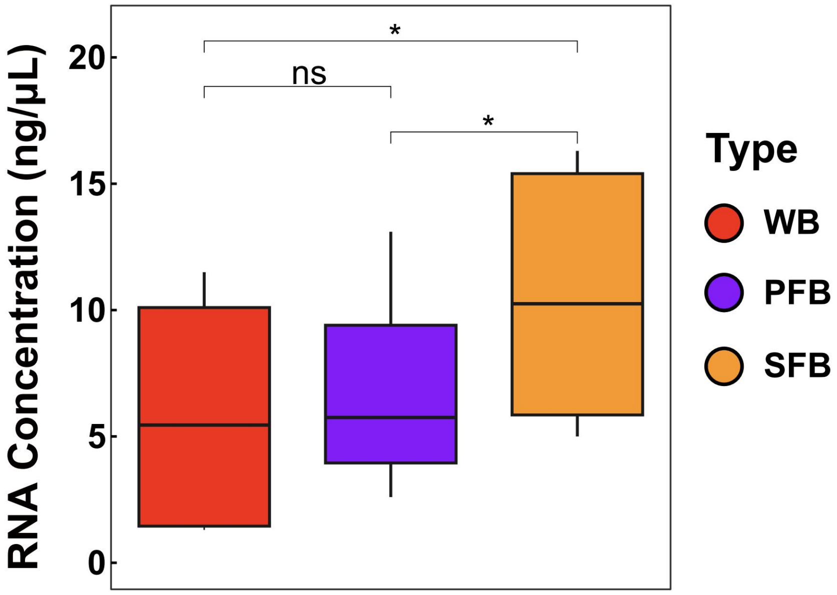

Fig S1. Normalized RNA concentration

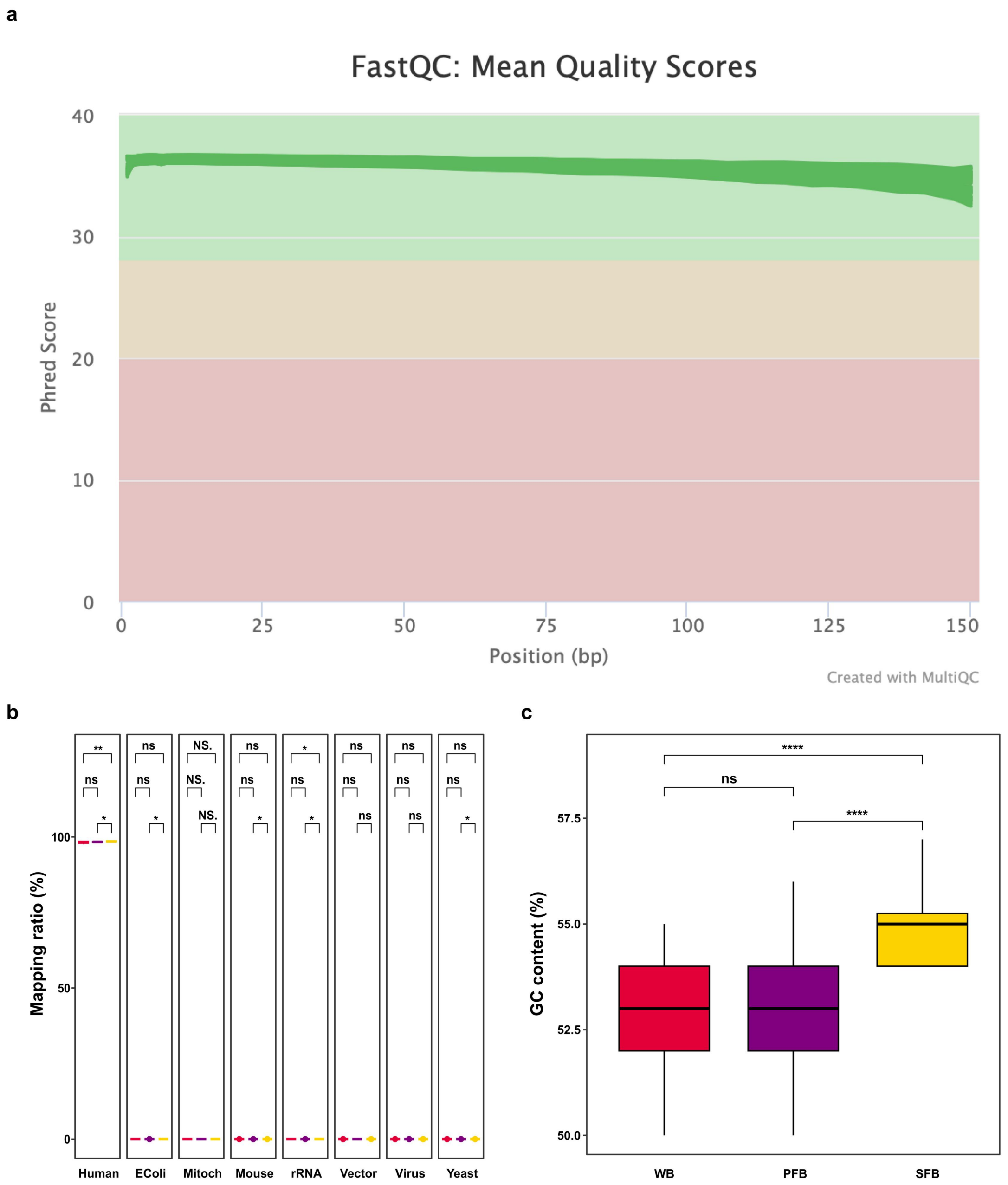

Fig S2. Quality control for clean data and sequence alignment

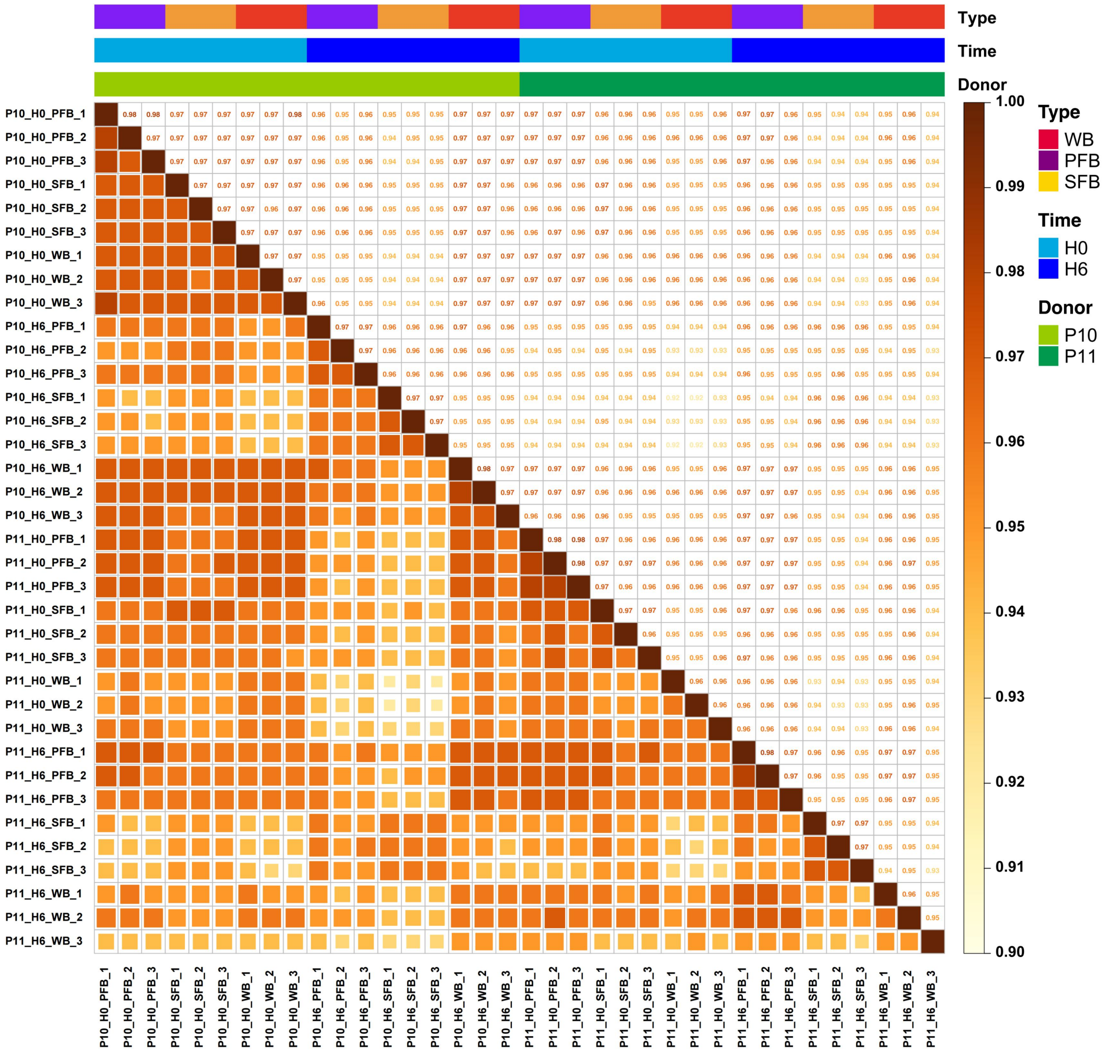

Fig S3. Pearson correlation coefficient analysis

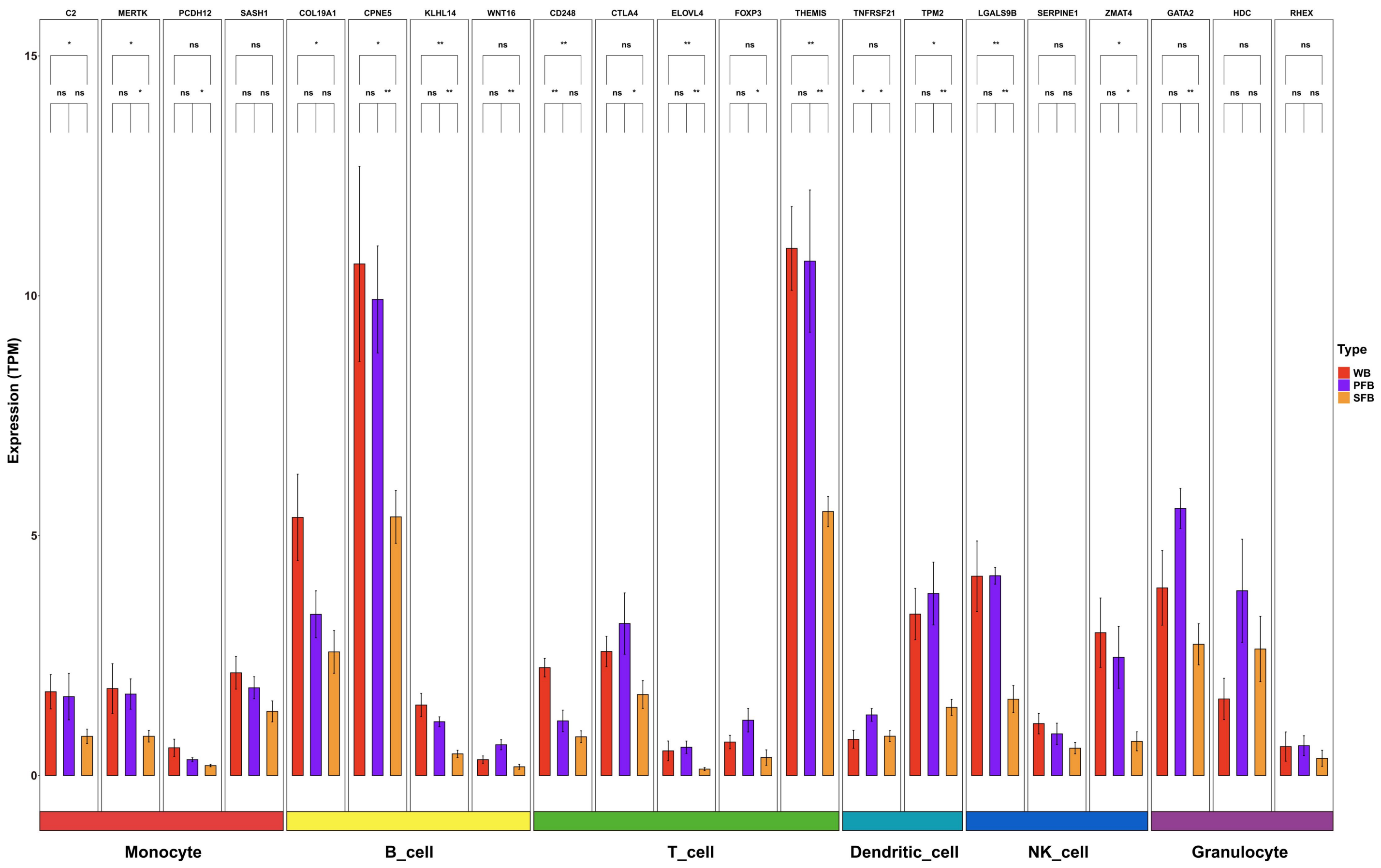

Fig S4. Expression of 21 immune cell-specific genes
